# Evaluation of the Influence of Specific Surface Treatments of RBA on a Set of Properties of Concrete

**DOI:** 10.3390/ma9030156

**Published:** 2016-03-03

**Authors:** Marcela Ondova, Alena Sicakova

**Affiliations:** Department of Material Engineering, Institute of Environmental Engineering, Faculty of Civil Engineering, Technical University of Kosice, Vysokoskolska 4, 042 00 Kosice, Slovakia; marcela.ondova@tuke.sk

**Keywords:** construction and demolition waste, recycled brick aggregate, impregnation, effective water/cement ratio, concrete properties

## Abstract

High water absorption of recycled brick aggregate (RBA) is one of the most discussed parameters in terms of its application in the production of concrete—its influence on the amount of mixing water and, hence, the quality of the concrete, is usually considered negative. In this paper, different methods of decreasing the absorption of RBA and, consequently, the impact on the properties of concrete, are described. The RBA has been treated to decrease the water absorption capacity by impregnation approach using specific impregnators. Afterwards, the RBA samples have been dried at two different temperatures in the laboratory oven—20 and 90 °C. Concretes using 4/8 fraction of the treated RBA instead of natural aggregate (NA) have been mixed and tested. The effectiveness of the RBA treatments have been evaluated on the basis of their influence on the properties of the hardened concrete; by means of the following tests: flexural strength, compressive strength, capillarity, total water absorption capacity, depth of water penetration under pressure, and frost resistance. The method of ranking by ordinal scale has been used as it is suitable for the comparison of a large set of results, while results have been analyzed in terms of the most important technological parameter that influences the quality of the concrete-effective water content. Out of all the tested surface-treatments of RBA, treatment by sodium water glass has the best potential for reduction of the water/cement (w/c) ratio. When the effective w/c ratio is kept within standard limits, concretes containing treated RBA are possible to be specified for various exposure classes and manufacturing in practice. The experiment confirms that at a constant amount of mixing water, with decreasing water absorption of RBA, the effective amount of water in the concrete increases and, hence, the final properties of the concrete decrease (get worse). As the water absorption of the RBA declines, there is a potential for the reduction of the w/c ratio and improvement in the quality of the concrete.

## 1. Introduction

The rapid growth in population has given rise to problems in terms of urban planning. Due to modern requirements for living quarters and the developed construction industries, old buildings are being demolished and replaced with new and modern ones [1]. In attempt to increase environmental sustainability, better ways to manage Construction and Demolition Wastes (C&DW) are being explored. One way to recycle the C&DW is by crushing it into aggregates. Depending on the recycled aggregate’s (RA) physical properties, it can be used in a variety of construction applications, thus reducing the environmental impact of the new construction [2,3]. The advantage of this method is that a significant amount of natural resources can be saved if the demolished materials of old buildings are recycled for use in new constructions [4].

One of the materials having a high potential for re-use as a replacement for NAs is old brick. A few studies on the effectiveness of using crushed clay-brick as a coarse aggregate in making concrete were reported in 1983–1999 [5,6,7]; however, the issue is still of interest today. Several studies monitored the properties of recycled bricks (e.g., cleanliness, porosity, high water absorption) for the purpose of improving the concrete. In terms of “cleanliness” of the recycled aggregate (RA), Yang *et al.* (2011) reported that the main problem of crushed brick is their origin. These clay bricks come from either load-bearing masonry walls or from cladding or party walls. It is not only costly but also technically impossible to completely separate concrete materials and recycled brick aggregate (RBA). Therefore, RBA is sometimes also called RA [8]. Schwerin *et al.* (2013) deal with recycled brick masonry aggregate (RBMA) and observe that approximately one-third of the RBMA is comprised of mortar (both in terms of weight and volume). It is likely that this large amount of mortar affects the performance of the RBMA in concrete mixtures [3].

Another study, dealing with RBA porosity, showed that the components used in the manufacture of concrete are the cause of poor performance in terms of compressive strength, modulus of elasticity, shrinkage, and resistance to freeze-thaw cycles [9,10]. Otoko (2014) indicates that RBA has a coefficient of porosity of 16.75%–24.50%, which is about 18% higher than the NA [11]. This fact was also confirmed in a subsequent study [12,13]. Another important parameter of RBA that is monitored in the long-term is water absorption. In 2008, Kesegić *et al.* found that water absorption and void content levels of RBA were several times higher than those of crushed stone aggregate [14]. The importance of this factor was also confirmed in a study by García-González *et al.* (2014). They discovered that the high water absorption of the RAs reduces the quality of the recycled concrete as it has a negative impact on the consistency, workability, and mechanical and durability properties of the final product. This is because the RA partially absorbs the water intended for cement hydration [15]. Paul and Dey (2014) indicate that the average water absorption is about 11.60% [16]. Zhang and Zong (2013) also confirm the value of water absorption of 16.58%. For comparison, the value of NA is about 1.45% [17]. Another study reported a coefficient of water absorption of 15.5% to 18.3% depending on the grain size of the RBA [18].

Aliabdo *et al.* (2014) state that the use of RAs reduces the overall unit weight of concrete masonry units. In fact, the overall unit weight of the subject units gradually decreases with an increase in the content of RBA. Based on their experimental evidence, it can be stated that completely replacing the fine and coarse aggregate with RA reduces the dry unit weight of concrete masonry units by up to 25% [18]. Similarly, many other works demonstrate the impact of RBA on the characteristics of concrete (e.g., [19,20,21]). The impact of the limiting factors mentioned above on the final properties of concrete has also been researched.

Other parameters (compressive strength, flexural strength, density, slump, tensile strength, modulus of elasticity, and specific gravity), and durability characteristics of concretes containing RBA were also studied in comparison with concretes containing NA in many works [8,22,23]. Yang *et al.* [8] state that NA produces stronger concrete than RBA does. It has been observed that as the substitution level of RBA increases, the compressive strength decreases by 3.1 MPa in seven days and 7.7 MPa in 28 days. These published results are based on a maximum 50% replacement of NA with RBA [8]. The paper by Bolouri Bazaz and Khayati [23] presents an overview study of RBA that characterizes the dependence of individual properties. This shows that the average water absorption levels for crushed brick and NA are about 28% and 1.5%, respectively. The authors describe this significant difference as the main reason for the reduction in the strength and durability of concrete made with crushed brick. They also observe a low specific gravity of brick compared to NA. It follows that the concrete made with crushed bricks is lighter than normal concrete. The compressive strength, the indirect tensile strength, and the flexural strength of all the recycled concrete mixes under investigation (with different curing and sample ages) have also been discussed in a paper by Afify and Soliman [24]. They report that recycled brick, when used as a replacement for coarse aggregate in concrete, without any admixtures, at the constant amount of cement and w/c ratio, causes decrease in the compressive strength, in the indirect tensile strength and in the flexural strength compared to normal concrete. The increasing w/c ratio causes the compressive strength, indirect tensile strength, and flexural strength to decrease. The authors also mention that the lower density of the recycled concrete leads to a higher sound and heat insulation. An extensive investigation was carried out with regard to the recycling of demolished brick aggregate concrete as coarse aggregate in a study by Mohammed *et al.* [25]. The concrete specimens on partial replacement (10%–50%) with RBA were tested at 7, 14, and 28 days for compressive strength, tensile strength, and Young’s modulus, and the results were compared with those of NA. It was revealed that RBA can be used as a coarse aggregate for making concrete of strength ranging from 20.7 to 31.0 MPa. This is consistent with the results of Liu and Zhang [26] who found that the strength grade of recycled concrete that uses bricks as coarse aggregate can reach C20 and C25 (compressive strength levels at 28 days are 21.2 and 27.55 MPa respectively). However, it is necessary to note that, according to the research results of Cao *et al.* [27], the compressive strength of recycled concrete brick decreases when the RA substitution ratio exceeds 50%.

It is clear that the research in the area of value of RBA by application to concrete is rather intensive. Two of the most discussed parameters of RBA are its high absorption and its influence on the amount of water in concrete and, hence, the quality of the concrete, which is usually considered negative. However, when defining the mixture proportions, it is necessary to evaluate this in the exact technological context. This research study presents the results of different methods of decreasing the absorption of RBA and its impact on concrete properties. The significance of this research study lies in testing the surface treatments of RBA with a specific approach, confirming the relation between absorption of RBA and effective water content, showing their impact on a relatively wide scale of concrete properties, and confirming the potential for reduction of w/c ratio in order to improve concrete quality. The method of ranking by ordinal scale has been used, as it is suitable for comparison of larger set of results. Results are analyzed in the context of the most important technological parameter that influences the quality of the concrete-effective w/c ratio. Results are also discussed in terms of economic impact and whether the [28] criteria are met.

## 2. Materials and Methods

### 2.1. Recycled Brick Aggregate (RBA) Treatments

**Figure 1:**
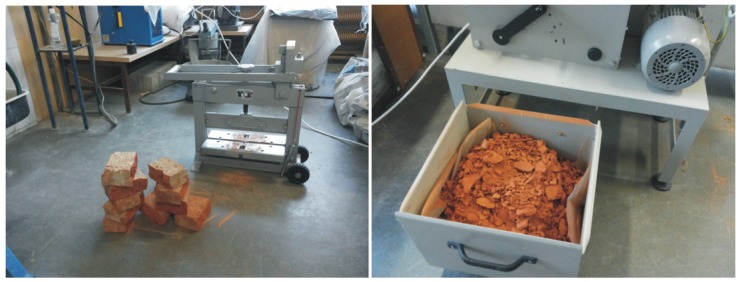
Processing the used bricks by laboratory techniques.

**Figure 2:**
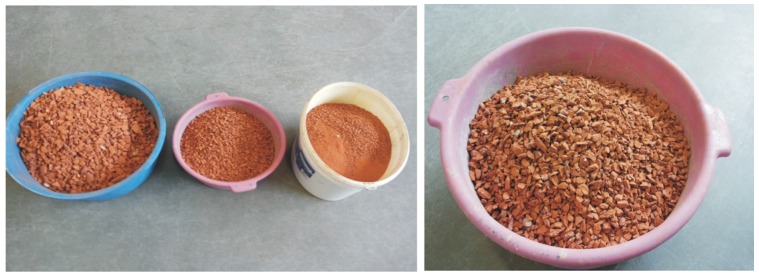
Sorted crushed brick and related 4/8 fraction.

For the experiment, 4/8 fraction of the RBA (cleaned of mortar) was prepared first by crushing and sorting, using laboratory techniques (Figure 1 and Figure 2). Bricks from a 60-year-old building were used as the input material. The total water absorption capacity (*WA_tot_*) was found to be 25.3%.

**Figure 3:**
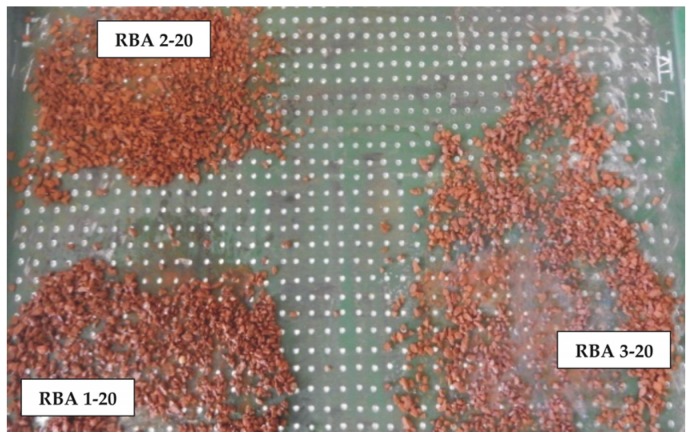
Treated recycled brick aggregate (RBA) samples after drying at 20 °C.

The RBA was treated to decrease the water absorption capacity by using the impregnation approach. For the sake of simplicity, impregnation was done under standard conditions, *i.e.*, immersion into the impregnator for 24 h at the ambient temperature of 20 °C. The RBAs were then dried in the laboratory oven by two different methods at 20 and at 90 °C, respectively. The first method of drying was intended as the most simple and energy efficient one, via simulation of air-drying under ambient conditions. Drying at 90 °C was intended as a way to retain the dry matter of the impregnator along with the minimum portion of water inside the pore system of RBA, *i.e.*, simulation of natural moisture of aggregates in real conditions. The treatment methods are shown in Table 1. Here, 1–3 are the impregnator types while 20 and 90 depict the treatment temperatures. Aggregate samples dried at 20 °C are shown in Figure 3.

### 2.2. Concrete Mixtures with RBAs

Concretes using 4/8 fraction of treated RBAs instead the NAs were mixed and tested. The composition is given in Table 2.

The experiment uses a constant amount of mixing water (*W_tot_*) in all mixtures. Due to the different water absorption capacities of RBAs, varying amounts of water form a part of the cement paste, resulting in the varying properties of hardened concrete. Here, it is important to differentiate between the w/c ratio expressed as the proportion of total mixing water to cement (it was 0.57 for all mixtures) and the “real”, *i.e.*, the effective w/c ratio (*W_ef_*/*C*) expressed as the effective proportion of water to cement (it has been found to be different for individual mixtures, see Table 3).

To identify the effective amount of water, the total amount is reduced by the amount to be absorbed by the RBA during the mixing of concrete. The first 10 min are critical for this process. Therefore, as a technological parameter, the short-term water absorption capacity in 10 min (*WA*_10_) was measured after the treatment process and the effective w/c ratio was expressed for each mixture. The effective w/c ratio was calculated by using Equation (1):

(1) $$\frac{W_{ef}}{C}=\frac{W_{tot}-WA_{10}\times m_{RBA}}{m_{C}}$$

where *W_tot_* is the total amount of mixing water (L), *WA*_10_ is the water absorption capacity (%) in 10 min, *m_RBA_* is mass of RBA (kg), and *m_c_* is mass of cement (kg).

The calculated *WA*_10_ of RBA and the corresponding effective w/c ratio *W_ef_*/*C* of concrete mixtures based on treated RBA (CRBA) are given in Table 3. Values up to 0.53 are standard for concretes made in accordance with [28].

During the mixing, consistency of concretes was also measured and evaluated. A direct dependence between the absorption capacity of RBA and consistency was observed. Results are presented and discussed in [29].

### 2.3. Methods of Testing the Properties of Hardened Concrete

The effectiveness of the RBA treatments is evaluated in terms of its influence on the properties of hardened concrete tested after 28 days of setting and hardening by the following tests:

- Flexural strength (MPa)
- Compressive strength (MPa)
- Capillarity due to capillary moisture content *m*_c_ after 90 min
- Total water absorption capacity (%)
- Depth of water penetration under pressure (mm)
- Frost resistance (frost resistance coefficient, expressed as a percentage of strength before testing) (%)

All these tests were performed in accordance with the relevant European standards.

#### 2.3.1. Flexural Strength

The standard test according to [30] (Testing hardened concrete. Flexural strength of test specimens) involves subjecting prismatic samples to a bending moment by the application of load through the upper and lower rollers. The center-point loading arrangement of the test was used here. The maximum load sustained is recorded. The flexural strength *f_cf_* (MPa) is calculated using Equation (2):

(2) $$f_{cf}=\frac{3F\times l}{{2d_{1}\times d_{2}}^{2}}$$

where *F* is the maximum load at failure, *l* is the distance between the supporting rollers (mm), and *d*_1_ and *d*_2_ are the lateral dimensions of the specimen.

#### 2.3.2. Compressive Strength

The Standard test according to [31] (Testing hardened concrete. Compressive strength of test specimens) generally involves using cubical samples with a size of 150 mm × 150 mm × 150 mm. Prepared specimens are tested with a compression testing machine after 28 days of curing. Load is applied gradually until the specimens are destroyed. Afterwards, the compressive strength of concrete *f_c_* (MPa) is determined according to the Equation (3):

(3) $$f_{c}=\frac{F}{A_{c}}$$

where *F* is maximum load at failure (N) and *A_c_* is the cross-sectional area of the specimen, on which the compressive strength acts (mm^2^).

#### 2.3.3. Capillary Moisture Content

The standard test under [32] (Methods of test for mortar for masonry. Determination of water absorption coefficient due to capillary action of hardened mortar) uses prismatic samples with a size of 40 mm × 40 mm × 160 mm. After drying to constant mass, one face of the specimens is immersed in water at a depth of 5–10 mm for a specific period of time. The increase in mass is determined. Standard periods are 10 and 90 min. For this experiment, measurements are done at the following intervals: 5, 10, 20, 30, 40, 50, 60, 70, 80, and 90 min. Capillarity is characterized by capillary moisture content *m_c_* according to Equation (4):

(4) $$m_{c}=A_{w}\times \sqrt{t}$$

where *A_w_* is absorption coefficient (kg/m^2^·h^1/2^) and *t* is time (h).

#### 2.3.4. Water Absorption Capacity

The standard test under national standard [33] (Determination of moisture content, absorptivity, and capillarity of concrete) is a trivial gravimetric method, wherein samples are dried at 105 °C and, then immersed in water up to constant mass. Absorption capacity is determined according to Equation (5):

(5) $$WA=\frac{m_{s}-m_{d}}{m_{d}}\times 100$$

where *WA* is water absorption capacity, *m_s_* is mass of saturated samples (g), and *m_d_* is the mass of the dry sample (g).

#### 2.3.5. Depth of Penetration of Water under Pressure

The standard test under [34] (Testing hardened concrete. Depth of penetration of water under pressure) uses cubical samples with a size of 150 mm × 150 mm × 150 mm. The specimens are placed into the apparatus and a water pressure of 500 kPa is applied for 72 h. The sample is then split in half and water penetration can be clearly seen and measured.

#### 2.3.6. Frost Resistance

Testing under [35] (Testing hardened concrete. Freeze-thaw resistance. Scaling) has three possible methods, of which the cube test was selected. Cubic samples with a size of 100 mm × 100 mm × 100 mm are used in this test. Test cubes, which are completely immersed in deionized water, are stressed by repeated freeze-thaw cycles. Freeze-thaw resistance is evaluated by determining the percentage mass loss of the test cubes after a specific number of cycles. In our test, 25 cycles were applied and evaluated in terms of percentage of initial strength before testing.

## 3. Results and Discussion

The experiment is based on the hypothesis, that lower absorption of the RBA and, hence, the higher effective w/c ratio should result in worse properties of hardened concrete, in accordance with general knowledge about negative influence of high amount of water on the properties of hardened cement stone. For optimization of the recipes for concrete with RBA, this information can be used to express a potential for the decrease of w/c ratio. This hypothesis should lead to connections between absorption of the RAs *WA*_10_ and the properties of the concrete.

The objective results of testing the properties of hardened concretes manufactured with 100% replacement of 4/8 fraction for treated recycled brick are presented in Table 4, which also shows standard deviations. These are the average results obtained after the usual 28 days of setting and hardening of three samples for each property.

The key properties of concretes are generally discussed in terms of standard [28] in relation to exposure classes. The fulfilment of technical conditions for the different exposure classes creates the conditions needed for the good performance of concrete, good durability, and long life. The standard classifies the environmental actions into the following exposure classes: X0—no risk of corrosion or attack; XC—corrosion induced by carbonation; XD—corrosion induced by chlorides other than from sea water; XS—corrosion induced by chlorides from sea water; XF—freeze-thaw attack with or without de-icing agents; and XA—chemical attack. The degree of aggressiveness is distinguished by numbers. For the specification of a certain exposure class, limits for both the compressive strength, and *W_ef_/C* should be taken into account (while the limit of cement amount is kept). For some classes, requirements for properties are set, as well. Based on our scope of tested properties and respective results, the suitable exposure classes for individual samples are given in Table 5, in order of decreasing strength. Total water absorption capacity is only considered for exposure class XA with a 4% limit—only CRBA 3–90 meets this limit. The depth of water penetration is considered for exposure classes XA and XF with a 50 mm limit—all samples meet this limit. Frost resistance coefficient is not required by [28]; our results after 25 freezing-thawing cycles can be generally considered as promising. Based on this assessment, it can be concluded that tested concrete samples containing treated RBA are possible to be specified for various exposure classes and manufacturing in practice.

The results of the tests were also evaluated by ranking on the ordinal scale; the resulting parameters were allocated points. As seven types of samples were assessed, the scale was 1–7 (best–worst). The value 1 was allocated to the best (lowest) absorption of the RA and to the best values of the concrete properties. For complex evaluation, the average value of concrete properties was calculated. Hence, the impact of the lower absorption of the RA on the quality of the concrete should appear in the final results vice-versa, *i.e.*, the best (lowest) absorption of RBA corresponds to the lowest quality concrete. The highest placement on the scale means the best potential for further decreasing the amount of mixing water and overall improvement of concrete quality. Table 6 shows the final ranking by the lowest *WA*_10_ and the respective treatment of RBA/mixture of CRBA.

When commenting on the final order of the samples of treated RA and respective concretes, it can be stated a relatively good consistency of experiment and *vice*-*versa* was achieved, especially in the marginal values, *i.e.*, the lowest absorption of the RBA gave the worst results in concrete properties (1–20 and 1–90) and the highest absorption gave the best values in concrete properties (3–90 and 0).

The best treatment of RBA with regards to decreasing the absorption is 1–20 (treatment by impregnation using pure sodium water glass and drying at 20 °C). The outcome is the highest effective w/c ratio and the worst concrete properties among all the tested samples. The worst treatment of the recycled material in terms of decreasing the absorption is 3–90 (impregnation by silanes/siloxanes emulsion and drying at 90 °C). The outcome is the lowest effective w/c ratio and the best concrete properties among all the tested samples.

In principal, the lowest absorption is achieved with RA dried at 20 °C (order being 1, 3, 4) due to the presence of residual water from the acting impregnator in the porous system, with the exception of RA 1–90 where drying at 90 °C leads to the second best result in terms of absorption. This principally corresponds to the results of the respective concrete in order 5.

On the other hand, using both impregnators, *i.e.*, the sodium water glass/water in a proportion of 1:1 and silanes/siloxanes emulsion (samples 2–90 and 3–90), and drying at 90 °C do not result in significantly lower absorption compared to sample without any treatment (0). The amount of dry content from the impregnators left in the porous system after drying is minimum and, hence, such treatment is of no importance from the technological point of view.

**Figure 4:**
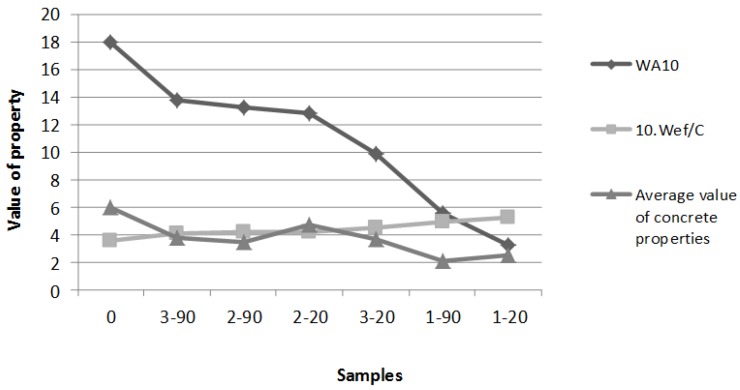
Relationships between water absorption capacity of RBA, effective amount of water in CRBA (concretes based on treated recycled brick aggregate), and final properties of CRBA.

For a summary, the relationships between water absorptivity of RBA, effective amount of water in concrete, and final properties of hardened concrete are shown schematically in Figure 4. Values of *WA*_10_, *W_ef_/C* (10× magnified proportionally due to be shown in actual scale of chart), and average properties of hardened concrete (from Table 4) were plotted into the chart in order of decreasing water absorption of RBA.

In Figure 4, the relationship resulting from the experiment methodology is clearly visible. With decreasing water absorption of RBA and increasing effective amount of water in the concrete, the final properties of concrete decrease (get worse). Higher values of absorption of the RA and relative decrease in w/c ratio lead to better concrete properties. However, in accordance with [36], it is necessary to also consider the influence on the other parameters of the concrete, such as durability due to quality of interfacial transition zone (ITZ). High porosity and absorption capacity of RA can lead to a porous microstructure of ITZ, along with the risk of cracks. With regard to this, it appears to be a better approach to improve surface properties of the RA by various treatments, therefore allowing for the exact regulation of w/c ratio. Various surface treatments of aggregates are also discussed in [36,37,38,39], and can be described briefly here as follows: pre-soaking, achievement of saturated conditions in dry surface before mixing, and surface coating. A generally favorable effect of the surface treatment of the RA on concrete properties is presented here.

Treated RAs can be a valuable material for building, bearing in mind the technical considerations, as well as sustainable and economical parameters. However, at present, the common point of view is that any waste treatments are too demanding technologically, energetically, and economically; recycling is preferred at it has lower additional costs. Hence, only the waste that is suitable for recycling by well-known methods are recycled. In other words, at the moment we can “choose” our waste and use only those that require only a small investment.

However, according to the [40], the member states have 70% quota for recycling their C&DW by 2020. With strict legislative pressure, the environmental aspect is getting prioritized and waste producers are gradually being forced to seek and use technologies that increase the recyclability of their waste. Of course, the treatment must still be technologically and energetically efficient as possible.

In terms of impregnation by immersion into a substance for treating surface properties, the highest costs are the substance cost and cost of energy for drying (not applicable if air dried). Considering current aggregate prices (for calculations, prices of regional supplier were used—0.70€/t including VAT (value added tax) for sorted brick waste of all fractions, and 16.60€/t including VAT for NA—4/8 fraction), the price difference creates a gap which allows for such treatments. Handling is not difficult and no special equipment or trained personnel are needed. Additionally, in [41], budgets for NAs processing and the treatment of RA using colloidal silica dispersion are calculated. Although with more expensive impregnation substance is used, they concluded that prices are comparable.

All activities in connection with waste treatments for use in concretes could be carried out in recycling facilities (companies) that could also employ people with less qualification and, therefore, help to reduce unemployment.

As seen from the results of this study, even easy and energy-friendly treatment (immersion—impregnation and air drying at ambient temperature, without any need for special equipment) offers a way to manufacture concretes that is comparable to NAs concrete.

## 4. Conclusions

The results can be summarized as follows:

1. The relation between water absorption of RBA, the effective amount of water, and set of concrete properties was confirmed: at a constant amount of mixing water, decline in water absorption of the RBA led to higher w/c ratio, and lower quality of concrete.
2. When applying RBA in various surface treatments, it is necessary to work with actual water absorption and the effective w/c ratio by adjusting the amount of mixing water to be absorbed by the RBA.
3. Out of all the tested surface treatments of the RBA, treatment by sodium water glass is the most effective in decreasing absorption. Therefore, it has the best potential for adjusting the mixing water and reduction of the w/c ratio, thus improving the quality of the concrete.
4. When surface treatments by impregnation are applied, it is better from a practical point of view to dry at lower temperatures, meaning part of the water stays in the porous system of the RBA.
5. Keeping the effective w/c ratio within standard limits, concretes containing treated RBA are possible to be specified for various exposure classes and manufacturing in practice.

## Figures and Tables

**Table 1:** Designation and description treatment methods of recycled brick aggregate (RBA).

Sample of RBA	Treatment Method
RBA 0	Control sample with no any treatment
RBA 1–20	Impregnator: Sodium water glass Drying: 20 °C
RBA 1–90	Impregnator: Sodium water glass Drying: 90 °C
RBA 2–20	Impregnator: sodium water glass and water, proportion 1:1 Drying: 20 °C
RBA 2–90	Impregnator: sodium water glass and water, proportion 1:1 Drying: 90 °C
RBA 3–20	Impregnator: hydrophobic emulsion of silanes and siloxanes Drying: 20 °C
RBA 3–90	Impregnator: hydrophobic emulsion of silanes and siloxanes Drying: 90 °C

**Table 2:** Composition of concrete mixtures based on treated RBA.

Component	Amount for 1 m^3^ of Ready-Mixed Concrete
Cement (kg/m^3^)	370
Natural aggregates fraction 0/4 (kg/m^3^)	1100
Treated RBA, fraction 4/8 (kg/m^3^)	425
Water total (L)	210
Polycarboxilate type of plasticizing admixture (%)	1.0

**Table 3:** Results of short-term water absorption capacity of treated RBA (*WA*_10_) and corresponding effective w/c ratio (*W_ef_*/*C*) of concrete mixtures Concrete based on treated recycled brick aggregate (CRBA).

Samples of RBA	*WA*_10_ (%)	Samples of Corresponding Concrete Mixture	*W_ef_/C*
RBA 0	18.0	CRBA 0	0.36
RBA 1–20	3.3	CRBA 1–20	0.53
RBA 1–90	5.6	CRBA 1–90	0.50
RBA 2–20	12.8	CRBA 2–20	0.42
RBA 2–90	13.2	CRBA 2–90	0.42
RBA 3–20	9.9	CRBA 3–20	0.45
RBA 3–90	13.8	CRBA 3–90	0.41

**Table 4:** Results of short-term water absorption capacity of treated RBA *WA*_10_ and corresponding properties of hardened concrete after 28 day of setting and hardening.

Samples RBA/CRBA	*WA*_10_		Flexural Strength		Compressive Strength		Capillary Moisture Content		Total Water Absorption Capacity		Depth of Water Penetration		Frost Resistance Coefficient	
	(%)	STDEV *	(MPa)	STDEV *	(MPa)	STDEV *	(kg/m^2^)	STDEV *	(%)	STDEV *	(mm)	STDEV *	(%)	STDEV *
0	18.0	0.4	9.0	0.6	45.3	1.3	2.9	0.6	6.0	0.3	12	0.2	101.7	0.4
1–20	3.3	0.6	7.1	0.5	32.7	0.8	4.0	0.4	8.0	0.7	33	0.7	95.7	0.9
1–90	5.6	0.7	7.4	0.8	33.8	0.8	3.8	0.4	7.6	0.7	13	0.3	106.2	0.4
2–20	12.8	0.5	6.8	0.3	31.4	0.8	4.1	0.9	6.6	1.0	20	0.3	132.1	1.1
2–90	13.2	0.4	4.3	0.5	41.4	1.2	2.6	0.4	5.1	0.6	23	0.5	102.4	0.4
3–20	9.9	0.8	5.7	0.1	23.0	1.0	3.6	0.3	5.4	0.8	22	0.2	99.1	0.7
3–90	13.8	0.5	7.8	0.8	37.8	0.6	2.6	0.3	3.9	0.6	14	0.4	109.5	0.5

* STDEV: Standard deviation.

**Table 5:** Exposure classes for individual samples in order of decreasing strength.

Samples RBA/CRBA	Strength Class	*W_ef_/C*		Exposure Class
		Limit	Actual	
0	C 35/45	0.45	0.36	all
2–90 3–90	C 30/37 C 30/37	0.55 0.50 0.45	0.42 0.41	XC 3, XD 1, XD 2, XF 1 XC 4, XS 1, XF 3 XF 4, XA 2
1–20 1–90 2–20	C 25/30 C 25/30 C 25/30	0.60 0.55	0.53 0.50 0.42	XC 2 XF 2
3–20	C 16/20	-	0.45	X 0

**Table 6:** The final ranking by the lowest *WA*_10_ and respective treatment of RBA/mixture of CRBA.

Order of *WA*_10_	Average Value of Concrete Properties	Respective Order of Concrete Properties	Sample of RBA/CRBA
1	6.00	7	1–20
2	3.83	5	1–90
3	3.50	3	3–20
4	4.70	6	2–20
5	3.75	4	2–90
6	2.10	1	3–90
7	2.50	2	0

## Abbreviations

The following abbreviations are used in this manuscript:

RBA: Recycled Brick Aggregate
C&DW: Construction and Demolition Wastes
RA: Recycled Aggregate
CRBA: Concrete based on treated Recycled Brick Aggregate
*WA_tot_*: Total water absorption capacity
*W_tot_*: Total mixing water
*WA*_10_: Water absorption capacity in 10 min
